# Supplementary material for: Cocaine self-administration induces sex-dependent protein expression in the nucleus accumbens
Source: Commun Biol. 2021 Jul 16;4:883. doi: 10.1038/s42003-021-02358-w (PMC8285523; doi:10.1038/s42003-021-02358-w)
Supplement: Supplementary file 2 — Supplementary Information [file 42003_2021_2358_MOESM2_ESM.pdf]

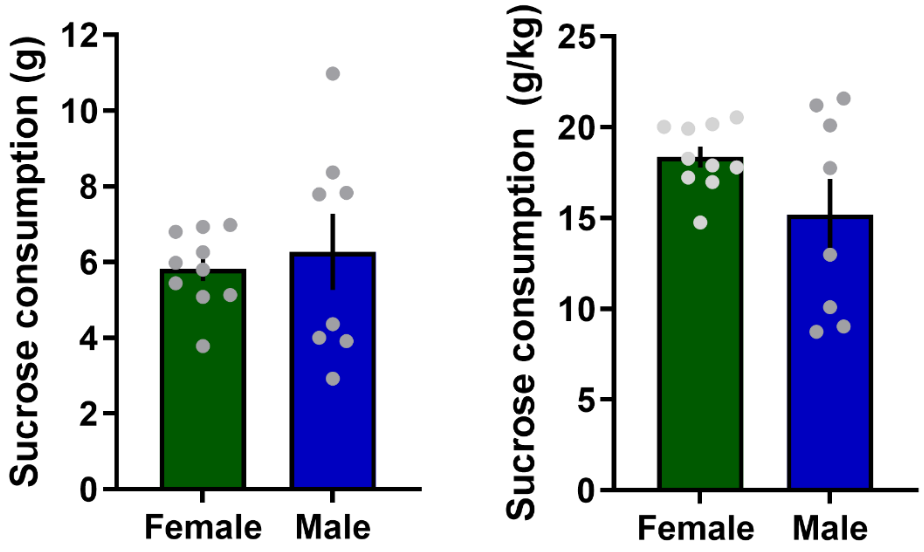

Supplementary Figure 1. Males and females consume similar amounts of sucrose on low effort schedules of reinforcement. On the last day of training on a fixed ratio 1 (FR1) schedule of reinforcement, males and females had unlimited access to sucrose. There were no sex differences observed in sucrose consumption between males and females when presented in total grams consumed (left) or as a function of body weight in each animal (right). One female was removed from analysis as a statistically significant outlier; however, this did not influence the results as they were non-significant whether the data point was included or not. Data reported as mean ± S.E.M.

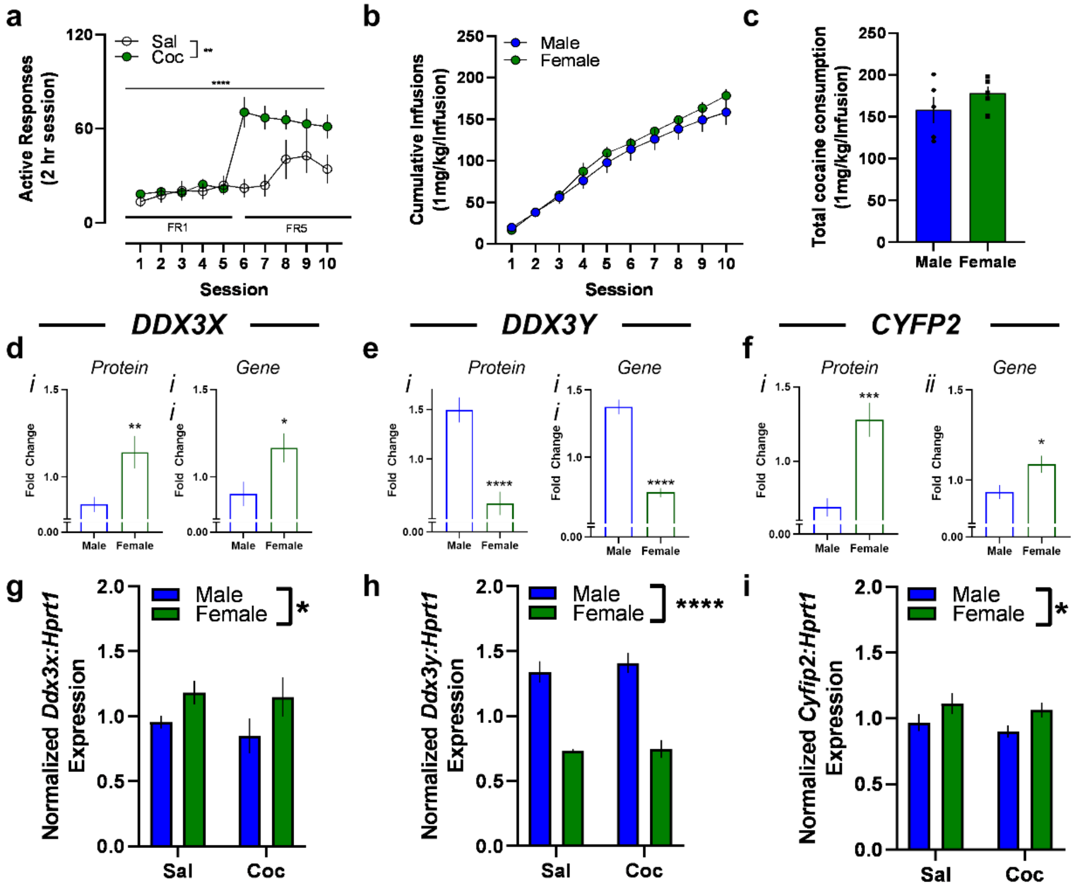

Supplementary Figure 2. Several proteomic differences are also identified at the transcriptional level. (**a**) A separate cohort of mice went through ten days of cocaine self-administration at 1 mg/kg/inj. Mice self-administering cocaine had higher responses on the active nose-poke compared to saline controls (two-way repeated measure ANOVA: main effect of Drug [$F_{(1,18)}$=8.46, p=0.009]; main effect of Session [$F_{(3.172,57.09)}$=14.51, p<0.0001]; significant Drug x Session Interaction [$F_{(9,162)}$=5.06, p<0.0001)]. (**b**) Male and female mice consume the same amount of cocaine across session. (**c**) Total cocaine consumption did not differ between males and females. (**d**) DDX3X (gene *Ddx3x)* was increased in females versus males at the (*i*) protein (Student's t-test, [$t_{(17)}$ = 2.79, p < 0.01]) and (*ii*) transcript (Student's t-test, [$t_{(18)}$ = 2.42, p < 0.05]) level. (**e**) DDX3Y (gene *Ddx3y)* was increased in males versus females at the (*i*) protein (Student's t-test, [$t_{(17)}$ = 5.48, p < 0.0001]) and (*ii*) transcript (Student's t-test, [$t_{(18)}$ = 9.96, p < 0.0001]) level. (**f**) CYFP2 (gene *Cyfip2*) was increased in females versus males at the (*i*) protein (Student's t-test, [$t_{(17)}$ = 4.41, p < 0.001]) and (*ii*) transcript (Student's t-test, [$t_{(18)}$ = 2.50, p < 0.05]) level. (**g**) Expression of *Ddx3x* is higher in the NAc of female mice compared to male mice (two-way ANOVA: main effect of Sex [$F_{(1,16)}$=5.37, p=0.034], but was not changed by cocaine experience. (**h**) Expression of *Ddx3y* is higher in the NAc of male mice compared to female mice (two-way ANOVA: main effect of Sex [$F_{(1,16)}$=91.11, p<0.0001], but was not changed by cocaine experience. (**i**) Expression of *Cyfip2* is increased in the NAc of female mice compared to male mice (two-way ANOVA: main effect of Sex [$F_{(1,16)}$=5.84, p=0.028], but was not changed by cocaine experience. Data reported as mean ± S.E.M.* p<0.05, ** p<0.01, *** p<0.001, ****p<0.0001. Sal = saline group, coc = cocaine group

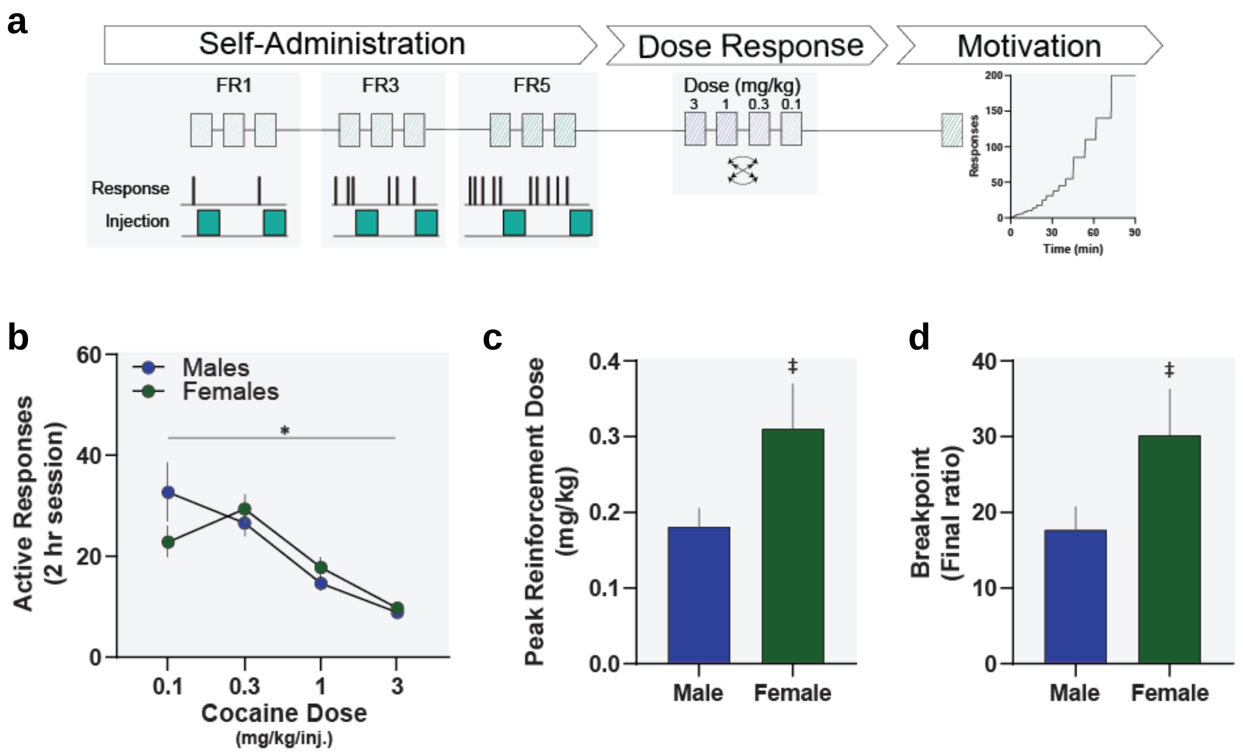

Supplementary Figure 3. Sex differences in consumption and motivation for cocaine. (**a**) A series of behavioral experiments were run to assess sex differences in motivation for cocaine self-administration in males and females. Schematic/timeline of self-administration. (**b**) Dose response curve for cocaine in male and female mice showing that the curve is significantly different between the sexes (two-way repeated measures ANOVA: main effect of Dose [$F_{(3,93)}$=25.00, p<0.0001]; significant Dose x Sex Interaction [$F_{(3,93)}$=2.81, p=0.04]). (**c**) The peak dose (dose with the highest response rates) for cocaine in male and female mice. Female mice show a trend towards a shift in their dose response curve where the peak (identified as the dose where responding is maximum) is shifted to a higher dose compared to males (Unpaired two-tailed t-test: t_{33}=1.88, p=0.0696) (**d**) Male and female mice went through a progressive ratio test of motivation at 0.3mg/kg/inj. Female mice showed a trend towards a higher break point compared to males indicating a higher level of motivation at this dose (Unpaired two-tailed t-test: t_{14}=2.04, p=0.060). Data reported as mean ± S.E.M. * p<0.05, ‡ p<0.1
