# Supplementary material for: Cocaine self-administration induces sex-dependent protein expression in the nucleus accumbens
Source: Commun Biol. 2021 Jul 16;4:883. doi: 10.1038/s42003-021-02358-w (PMC8285523; doi:10.1038/s42003-021-02358-w)
Supplement: Supplementary file 3 — Description of Additional Supplementary Files [file 42003_2021_2358_MOESM3_ESM.pdf]

Description of Additional Supplementary Files

File name: Supplementary Data 1-18

Description:

Supplementary Data 1: *Protein and Gene Identifiers.* Uniprot Entry/ID, accession number, and protein name of proteins identified in the nucleus accumbens (NAc) using data independent acquisition (DIA) mass spectrometry. Gene name conversions used for subsequent analyses are listed.

Supplementary Data 2: *Differentially Expressed Proteins in Males versus Females at Baseline (Fold Change of Male).* Uniprot ID of proteins identified to be differentially expressed at baseline in the NAc of male versus female mice. P-value and fold change reported as compared to male baseline NAc protein expression.

Supplementary Data 3: *Differentially Expressed Proteins in Female Cocaine versus Female Saline Controls.* Uniprot ID of proteins differentially expressed following cocaine self-administration in the NAc of female mice. P-value and fold change reported as compared to female saline control.

Supplementary Data 4: *Differentially Expressed Proteins in Male Cocaine vs. Male Saline Control.* Uniprot ID of proteins differentially expressed following cocaine self-administration in the NAc of male mice. P-value and fold change reported as compared to male saline control.

Supplementary Data 5: *Venn Comparison of Proteins Altered by Cocaine Self-Administration in Males and Females (Compared to Sex-Specific Saline Control).* Uniprot ID of proteins used for Venn diagram comparison in **figure 4b, *left***. Proteins regulated by cocaine self-administration in males only, females only, and in both males and females are listed.

Supplementary Data 6: *STRING Analysis.* Uniprot ID and associated gene name of proteins that make up the functional STRING network. Proteins identified in either Protein Binding, Neuron Projection, or both gene ontology (GO) terms are listed.

Supplementary Data 7: *Gene Ontology (GO) Analysis of Male versus Female at Baseline (Up in Female*)**.** GO terms identified from proteins upregulated in females (compared to males) at baseline, with relevant statistics.

Supplementary Data 8: *GO Analysis of Male versus Female at Baseline (Down in Female).* GO terms identified from proteins downregulated in females (compared to males) at baseline, with relevant statistics.

Supplementary Data 9: REVIGO Analysis (Up in Female). REVIGO analysis of GO terms from proteins upregulated in Females (compared to males) at baseline, with relevant statistics. Redundant GO terms in Supplementary Data 7 were eliminated using REVIGO.

Supplementary Data 10: REVIGO Analysis (Down in Female). REVIGO analysis of GO Terms from proteins downregulated in females (compared to males) at baseline, with relevant statistics. Redundant GO terms in Supplementary Data 8 were eliminated using REVIGO.

Supplementary Data 11: GO Analysis of Female Cocaine versus Female Saline (All Proteins). GO Terms identified from proteins significantly different in female cocaine compared to female saline, with relevant statistics.

Supplementary Data 12: GO Analysis of Male Cocaine versus Male Saline (All Proteins). GO Terms identified from proteins significantly different in male cocaine versus male saline groups, with relevant statistics.

Supplementary Data 13: REVIGO Analysis of Female Cocaine versus Female Saline (All Proteins). Redundant GO Terms in Supplementary Data 11 were eliminated using REVIGO.

Supplementary Data 14: REVIGO Analysis of Male Cocaine versus Male Saline (All Proteins). Redundant GO Terms in Supplementary Data 12 were eliminated using REVIGO.

Supplementary Data 15: RRHO Ranked List of Male Cocaine versus Male Saline (Log2 Fold Change). Uniprot ID of proteins significantly altered in male cocaine compared to male saline. Rank ordered list was generated and ranked based on Log2 fold change.

Supplementary Data 16: RRHO Ranked List of Female Cocaine versus Female Saline (Log2 Fold Change). Uniprot ID of proteins significantly altered in female cocaine versus female saline. Rank ordered list was generated and ranked based on Log2 fold change.

Supplementary Data 17: RRHO Ranked List Male versus Female Baseline (Log2 Fold Change). Uniprot ID of all proteins identified in the NAc using DIA mass spectrometry. Rank ordered list was generated and sorted based on Log2FC of male proteins compared to female controls at baseline (between saline groups).

Supplementary Data 18: RRHO Ranked List Female versus Male Baseline (Log2 Fold Change). Uniprot ID of all proteins identified in the NAc using DIA mass spectrometry. Rank ordered list was generated and sorted based on Log2FC of female proteins compared to male controls at baseline (between saline groups).
