# Supplementary material for: Cocaine self-administration induces sex-dependent protein expression in the nucleus accumbens
Source: Commun Biol. 2021 Jul 16;4:883. doi: 10.1038/s42003-021-02358-w (PMC8285523; doi:10.1038/s42003-021-02358-w)
Supplement: Supplementary file 5 — Reporting Summary [file 42003_2021_2358_MOESM5_ESM.pdf]

nature research

Corresponding author(s): Calipari ES

Last updated by author(s): Jun 15, 2021

Reporting Summary

Nature Research wishes to improve the reproducibility of the work that we publish. This form provides structure for consistency and transparency in reporting. For further information on Nature Research policies, see our Editorial Policies and the Editorial Policy Checklist.

Statistics

For all statistical analyses, confirm that the following items are present in the figure legend, table legend, main text, or Methods section.

n/a	Confirmed	
☐	☒	The exact sample size (*n*) for each experimental group/condition, given as a discrete number and unit of measurement
☐	☒	A statement on whether measurements were taken from distinct samples or whether the same sample was measured repeatedly
☐	☒	The statistical test(s) used AND whether they are one- or two-sided<br>*Only common tests should be described solely by name; describe more complex techniques in the Methods section.*
☐	☒	A description of all covariates tested
☐	☒	A description of any assumptions or corrections, such as tests of normality and adjustment for multiple comparisons
☐	☒	A full description of the statistical parameters including central tendency (e.g. means) or other basic estimates (e.g. regression coefficient) AND variation (e.g. standard deviation) or associated estimates of uncertainty (e.g. confidence intervals)
☐	☒	For null hypothesis testing, the test statistic (e.g. *F*, *t*, *r*) with confidence intervals, effect sizes, degrees of freedom and *P* value noted<br>*Give P values as exact values whenever suitable.*
☒	☐	For Bayesian analysis, information on the choice of priors and Markov chain Monte Carlo settings
☒	☐	For hierarchical and complex designs, identification of the appropriate level for tests and full reporting of outcomes
☒	☐	Estimates of effect sizes (e.g. Cohen's *d*, Pearson's *r*), indicating how they were calculated

Our web collection on statistics for biologists contains articles on many of the points above.

Software and code

Policy information about availability of computer code

Data collection	Behavioral data was collected using MedAssociates V. Data-independent mass spectrometry was collected using Scaffold DIA software v. 1.1.1. RT-qPCR data was collected using CFX BioRad Maestro. All software used is further described in the Methods section.
Data analysis	Data was analyzed using Graphpad Prism 8.0. DMass spectrometry data was analyzed using Scaffold DIA proteomics software (v.2.2.1), gProfiler (v: e101_eg48_p14_baf17f0; open access), REVIGO (open access), GeneOverlap (R-script available on GitHub), RRHO2 (R-script available on GitHub), Microsoft Excel (v. Office 365). Portions of figures were created with BioRender.com. All additional code is further described in Methods section.

For manuscripts utilizing custom algorithms or software that are central to the research but not yet described in published literature, software must be made available to editors and reviewers. We strongly encourage code deposition in a community repository (e.g. GitHub). See the Nature Research guidelines for submitting code & software for further information.

Data

Policy information about availability of data

All manuscripts must include a data availability statement. This statement should provide the following information, where applicable:

- Accession codes, unique identifiers, or web links for publicly available datasets
- A list of figures that have associated raw data
- A description of any restrictions on data availability

Raw proteomic data generated from mass spectrometry for Figures 2, 4, and 5 has been uploaded to ProteomeXchange with identifier PXD022832.

Field-specific reporting

Please select the one below that is the best fit for your research. If you are not sure, read the appropriate sections before making your selection.

- [x] Life sciences
- [ ] Behavioural & social sciences
- [ ] Ecological, evolutionary & environmental sciences

For a reference copy of the document with all sections, see nature.com/documents/nr-reporting-summary-flat.pdf

Life sciences study design

All studies must disclose on these points even when the disclosure is negative.

Sample size	Sample size for mass spectrometry proteomic analysis and behavioral analyses were determined based on field standard for generating sufficient power for group-wise comparisons.
Data exclusions	Animals were excluded from analysis of the progressive ratio for failing to meet minimum performance criteria (determined a priori). Experimenters were blind to animal subject group for determining exclusion criteria.
Replication	Behavioral assays were run in multiple cohorts of approximately equal sample sizes (both cohort size and group sample size were comparable) Replication attempts were successful as findings were consistent across cohorts.
Randomization	Group subjects were age-matched, however, randomization based on sex is not needed for this study as sex is an independent variable of the study.
Blinding	Investigators were not blinded to subject group (divided by male versus female) during behavioral assays as animal sex can be determined visually during standard rodent handling procedures. Investigators were blind to subject group of samples during mass spectrometry data collection and processing.

Reporting for specific materials, systems and methods

We require information from authors about some types of materials, experimental systems and methods used in many studies. Here, indicate whether each material, system or method listed is relevant to your study. If you are not sure if a list item applies to your research, read the appropriate section before selecting a response.

Materials & experimental systems

n/a	Involved in the study	
x		Antibodies
x		Eukaryotic cell lines
x		Palaeontology and archaeology
	x	Animals and other organisms
x		Human research participants
x		Clinical data
x		Dual use research of concern

Methods

n/a	Involved in the study	
x		ChIP-seq
x		Flow cytometry
x		MRI-based neuroimaging

Animals and other organisms

Policy information about studies involving animals; ARRIVE guidelines recommended for reporting animal research

Laboratory animals	Male and female C57BL/6J mice were provided by Jackson Laboratory at 8-weeks old. 12-week old male and female Sprague Dawley rats were provided by Envigo.
Wild animals	The study did not involve wild animals.
Field-collected samples	The study did not involve field-collected samples.
Ethics oversight	All experiments were approved by the Institutional Animal Care and Use Committee of Vanderbilt University Medical Center and conducted according to National Institutes of Health guidelines for animal care and use.

Note that full information on the approval of the study protocol must also be provided in the manuscript.
